# Immune phenotypes of microglia in human neurodegenerative disease: challenges to detecting microglial polarization in human brains

**DOI:** 10.1186/s13195-015-0139-9

**Published:** 2015-08-19

**Authors:** Douglas G. Walker, Lih-Fen Lue

**Affiliations:** Laboratory of Neuroinflammation, Banner Sun Health Research Institute, 10515 West Santa Fe Drive, Sun City, AZ 85351 USA

## Abstract

Inflammatory responses in the brain, which can be demonstrated by changes in properties of microglia, the brain-resident macrophages, are a common feature of human neurodegenerative diseases. Different monocyte/macrophage phenotypes have been defined by changes in expression of cytokines, receptors and other markers as a response to different classes of stimuli. Monocytes, macrophages and microglia can have a range of phenotypes with associated properties depending on their microenvironment. Macrophage/microglia polarization states have been defined as classical activation (M1), alternative activation (M2a), type II alternative activation (M2b) or acquired deactivation (M2c). Available markers for identifying microglial phenotypes in human brains are still limited; those available provide incomplete information on the functions or polarization states of microglia observed in tissues from diseases such as Alzheimer’s disease, Parkinson’s disease and multiple sclerosis. The most widely used marker to describe activated microglia in human brains, particularly diseased brains, has been HLA-DR, the major histocompatibility complex II protein. HLA-DR-positive microglia can have a wide range of activation morphologies that are affected not only by disease pathology, but also by their differentiation states and brain regions. Two other widely used markers to identify microglia in human brains are ionized calcium binding adaptor molecule-1 and CD68. Although their expression changes in diseased brains, these markers do not show specificity for different phenotypes. Over the years there have been studies with additional markers that attempt to further define microglial properties, particularly in Alzheimer’s disease brains. Most studies have employed immunohistochemical techniques to identify microglia in tissue sections, but recent advances in this field have allowed gene expression profiling of microglia upon immediate isolation from brains. We will review which markers might better define different activation phenotypes of microglia in human brains and whether they fit into current microglial polarization schemes.

## Introduction

It has been more than a quarter-century since the “new era” of studies of inflammation in Alzheimer’s disease (AD) and Parkinson’s disease (PD) brains identified the major histocompatibility complex class II (MHC-II) protein HLA-DR as a marker to identify “activated” microglia. However, there are many features of microglia in human neurodegenerative diseases that remain to be understood [1–4]. Defining microglial properties in relation to neuropathology has generally required antibodies that can be used to identify different types of microglia in fixed human tissue sections using immunohistochemistry techniques. Recently, gene expression profiling and flow cytometry techniques of microglia isolated from brain or excised from tissue sections have also been applied to address these issues [5, 6].

Initial human neuropathology studies promoted the hypothesis that increased expression of HLA-DR by microglia, particularly if combined with a hypertrophic morphology and closely associated with pathological structures, identified cells presumed to be causing inflammatory damage—by current definition, being classically activated or having an M1 phenotype (reviewed in [2, 3]). Using appropriately fixed tissue samples and suitable monoclonal antibodies to HLA-DR, it was possible to demonstrate microglia with these morphologies associated with amyloid plaques and neurofibrillary tangles, the hallmark pathological structures of AD (reviewed in [2]), free neuromelanin and dopaminergic neurons in the substantia nigra (SN) of PD brains [7], or around demyelinated plaques in brains from multiple sclerosis (MS) cases [8]. Many additional studies have since used antibodies to HLA-DR to confirm these findings in AD, PD and MS brain tissues. HLA-DR-positive microglia have also been observed in pathology-rich brain regions in human neurodegenerative diseases such as dementia with Lewy bodies (DLB) and frontal temporal dementia (FTD) [9]. The close interactions of HLA-DR-activated microglia with pathological structures suggested that these abnormal protein structures were activating the microglia, and also that these activated microglia might be enhancing the pathological processes.

**Fig. 1:**
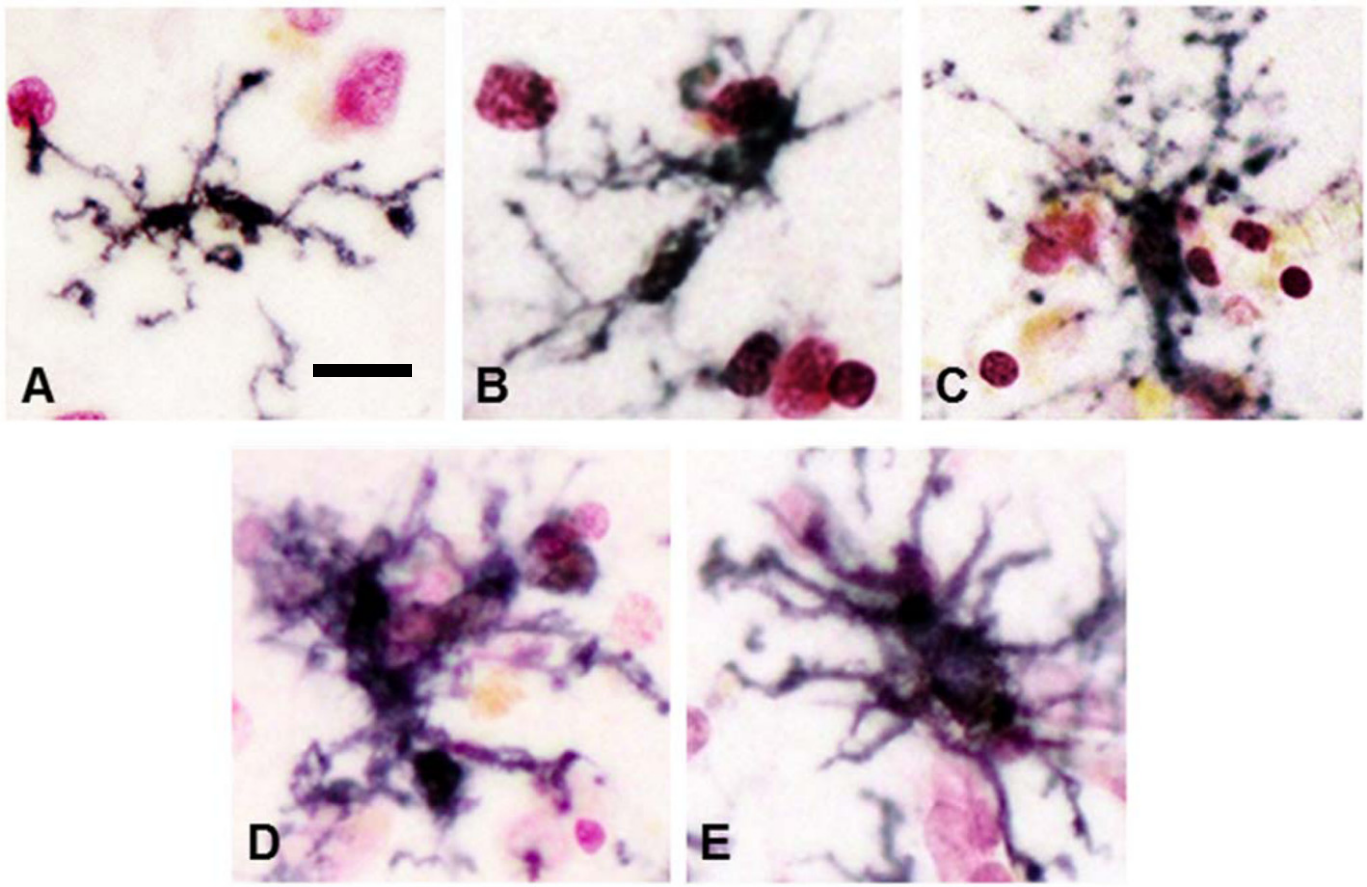
Various morphologies of microglia in human brain sections. Progressive changes in morphology of HLA-DR-expressing microglia in a pathology-rich section from an AD case. HLA-DR-expressing microglia can be found with various activation morphologies ranging from **a** highly ramified to **c** moderately hypertrophic to **e** highly activated with enlarged cell body and processes. **b**, **d** Intermediate changes in morphology. Sections were stained using antibody LN3 (1:1,000 dilution; Abcam, Cambridge, MA, USA) using nickel-enhanced diaminobenzidine peroxidase immunohistochemistry and counterstained with neutral red

Questions remain about the functional significance of HLA-DR expression by microglia. To illustrate this point, Fig. 1 shows representative examples of HLA-DR-positive microglia in a single AD temporal cortex section that have morphologies ranging from highly ramified (considered resting) to those with hypertrophic cell bodies (considered activated and inflammatory). Data now suggest that HLA-DR reactivity alone does not identify microglial polarity or function since HLA-DR upregulation can also be a feature of alternatively activated microglia/macrophages, which are microglia/macrophages with anti-inflammatory reparative phenotypes [10]. Many studies have been published presenting results of immunohistochemistry with a range of different antigenic markers identifying altered expression in microglia in human brains affected by disease processes (key reviews [2, 3]). These studies have substantially advanced the field of neuroinflammation; these markers will be considered in relation to the function of identified cells, as well as how they fit into the context of microglial polarization (Table 1).

**Table 1:** Selected immune function markers expressed by human microglia

Designation (references)	M1	M2	AD brains
CD14 [15, 16]	++	–	Vessels and around plaques
CD16, CD32, CD64 [13, 38]	Uncertain	++	Increased on phagocytic microglia
CD45 [39, 40]	++	+	All microglia: increase in disease
CD11b [49]	Uncertain	Uncertain	Most microglia: increase in disease
CD68 [20]	Uncertain	Uncertain	Increased on plaque microglia
CD163 [22]	–	++	M2c: vessel-associated microglia
CD206 [34]	–	mRNA perivascular macrophages	
CD209 [21]	–	++	M2a: MS lesion-associated microglia
CCL22 [21]	–	++	M2a: MS lesion-associated microglia
CD36 [18]	Uncertain	++	Phagocytic microglia
Cyclooxygenase-1 [43]	–	++	Plaque-associated microglia
TLR-2 [15]	++	+	Plaque-associated microglia
Ferritin [41]	++	Uncertain	Pathology-associated microglia
TREM2 [46]	–	+	Plaque-associated microglia
CD33 [45]	–	+	Increased expression in AD
CSF-1R [23]	Uncertain	Uncertain	Increased expression in AD

There is uncertainty about the assignment of the protein markers to M1 or M2. These are based on the authors’ interpretation of different literature including data from in vitro experiments. – to ++, observed changes in microglia in brain samples. Assignment based on in vitro findings. M2 includes M2a, M2b and M2c

*AD* Alzheimer’s disease, *CSF-1R* colony-stimulating factor-1 receptor, *MS* multiple sclerosis, *TLR* Toll-like receptor, *TREM-2* triggering receptor expressed by myeloid cells-2

## Defining immune phenotypes of microglia and macrophages

A range of profiling studies by Gordon and colleagues identified what could be valid markers for classically or alternatively activated human macrophages (key papers [11, 12]). A scheme was developed that divided microglia/macrophages into classically activated (M1) cells based on changes in responses to the proinflammatory agents lipopolysaccharide (LPS) and interferon gamma (IFN-γ). M1 activated microglia can produce reactive oxygen species as a result of reduced nicotinamide adenine dinucleotide phosphate (NADPH) oxidase activation (respiratory burst), and increased production of proinflammatory cytokines such as tumor necrosis factor (TNF) alpha and interleukin (IL)-1β. These are the types of microglia/macrophages that could be mediating inflammatory tissue damage.

Alternative activation (M2), as the other arm of this scheme, was defined as the phenotype of microglia/macrophages responding to IL-4 or IL-13; this is now known as M2a. Microglia with M2a phenotypes have increased phagocytosis and produce growth factors such as insulin-like growth factor-1 and anti-inflammatory cytokines such as IL-10 [10]. These types of microglia could be removing cellular debris and promoting tissue repair. The alternative activation scheme has been refined into two further subcategories: M2b and M2c. M2b (type II alternative activation) is induced by ligation of immunoglobulin Fc gamma receptors (FcγRs) (CD16, CD32 or CD64) by immune complexes on LPS or IL-1β primed microglia/macrophages, which results in downregulated expression of IL-12, increased IL-10 secretion and increased HLA-DR expression. This phenotype is also characterized by increased expression of CD32 and CD64, which has been described on microglia in AD brains [13], and associated with increased phagocytic activity. CD32 expression seems to be crucial for type II activation to occur in human monocytes and macrophages. M2c (acquired deactivation) can be induced by the anti-inflammatory cytokine IL-10 or glucocorticoids, with increased expression of transforming growth factor (TGF) beta, sphingosine kinase (SPHK1) and CD163, the membrane-bound scavenger receptor for haptoglobin/hemoglobin complexes [14].

## Microglia in human brains

Markers used to describe microglia in human brains include CD14 [15, 16], CD40 [17], CD16, CD32 and CD64 (the three classes of immunoglobulin Fc receptors) [13], phagocytic receptors (CD36) [18] and macrophage scavenger receptor MSR-A [19], CD68 (a lysosomal marker indicative of phagocytic activity of microglia, example [20]), CD74, CD86 and C–C chemokine ligand CCL22 [21], CD163 [22] and macrophage colony-stimulating factor-1 receptor (CSF-1R; CD115) [23]. How these markers might fit into the M1 or M2 classification scheme is suggested in Table 1. A common feature described for most of these markers has been increased expression on hypertrophic microglia associated with pathology; however, it is now appreciated that a whole spectrum of microglial phenotypes and morphologies can be present within a human brain (Fig. 1) [24]. Microglia can be at different stages of differentiation, activation and function in tissue, but currently used markers do not show these features.

CD40 has been consistently shown to be a marker for M1 activation of macrophages/microglia [17]. In AD brains, widespread expression of CD40 by microglia has been observed; however, it was noted that increased microglial CD40 expression was only prominent in AD brains with other disease factors such as bacterial encephalitis [17]. These findings do suggest that AD pathology alone might not be sufficient to induce M1 activation and significant CD40 expression. An interesting question remaining is what do M2 microglia look like in human brain tissue, and how different are they morphologically from M1 microglia? A recent study examining these concepts in MS brain tissue defined CD40, CD74 and C–X–C chemokine ligand CXCL10 as markers of M1 activation, and CCL22 and CD209 (DC-SIGN) as M2a markers [21]. In this study, mixed populations of microglia with both M1 and M2 phenotypes were observed in diseased brain tissue. For example, CCL22 immunoreactive microglia were also CD40-positive and HLA-DR-positive. The ability of microglia to transition between M1 and M2a phenotypes was demonstrated in vitro in this study. It is worth noting that this study could not demonstrate microglia immunoreactive for CD206, a prototypical M2a marker [21]. CD206 immunoreactivity was only observed in macrophages present in vessels. These findings indicate the need for validation of CCL22 as a genuine marker for M2a in human microglia.

In studies of M1 and M2 markers in RNA samples extracted from AD brains, coexpression of M1, M2a, M2b and M2c markers could be detected in samples [25, 26]. However, one limitation to this approach that affects all human tissue studies is that gene expression intensities might include mRNA from monocytes/macrophages present within the brain blood vessels. Comparing gene expression profiles of M1 and M2a human macrophages identified C–C chemokine receptor CCR7, IL2Ra, IL15Ra, chemokines CXCL11, CCL19, CXCL10 and CXCL9 and indoleamine-pyrrole-2,3-dioxygenase as the best M1 markers, with P2Y5 purinergic receptor, C-type lectin receptors DCL-1 and DECTIN1, CD209, macrophage mannose receptor and chemokines CCL13, CCL18 and CCL23 as markers of M2a alternative activation [12]. This landmark paper identified many markers for blood macrophages but indicated targets for study in human microglia. Antibodies to most of these markers have still not been tested to determine whether they identify microglia in human brains. One other marker identified in this study [12] is CD36, which has been studied in AD brains and identifies plaque-associated microglia. CD36 is a phagocytic scavenger receptor for amyloid beta peptide (Aβ) [18], but has been defined as an M2a alternative activation marker for human macrophages [12].

Another widely used marker for microglia in tissues is ionized calcium binding adaptor molecule-1 (IBA-1), which interacts with actin bundles and is involved with membrane ruffling and phagocytosis [27]. IBA-1 has the same sequence as allograft inflammatory factor-1 (AIF-1). This protein can be demonstrated in all microglia with some increase in expression and cellular rearrangement in activated microglia. Its applicability as an activation marker is still unresolved; a semiquantitative analysis of microglia in the SN of PD and incidental Lewy body disease (ILBD) cases compared with controls showed a slight increase in IBA-1 immunoreactivity, but a large progressive increase in CD68, a marker for actively phagocytic microglia [28]. As IBA-1 appears to identify all microglia, it is not useful for identifying their immune phenotype/polarity [27]. This was also demonstrated in tissue sections from AD cases that had received the Aβ peptide vaccine as treatment, which resulted in significantly increased phagocytosis of Aβ by microglia [29]. Comparing the microglia load (IBA-1 reactivity) between control and immunized cases showed no overall difference in microglial numbers, but, by contrast, microglial markers associated with phagocytosis (CD68, CD32, CD64 and macrophage scavenger receptor MSR-A) were significantly reduced in the immunized cases where Aβ removal had occurred [20]. These data demonstrate how levels of expression of certain markers can change depending on the functional state of microglia.

## Investigation of microglial phenotypes in human brains

The investigation of polarization markers expressed by microglia in the brain has been extended from immunohistochemistry techniques with antibodies to gene expression profiling and flow cytometry methodology of microglia directly extracted from human brains [6, 30–32]. Phenotyping studies of ex vivo brain isolated microglia have utilized postmortem human brain white matter in relation to studies of MS [6, 30] or surgical samples of temporal cortex resected as treatment for epilepsy [31]; however, these findings have relevance to all neurodegenerative diseases with microglial components. In one of these studies, ex vivo isolated microglia from white matter showed positive reactivity to HLA-DR, CD16, CD32 and CD64, but not to CD14, CD80, CD163, CD200 receptor (CD200R) or CD206 [6]. This result is not indicative of either an M1 or an M2a phenotype. Microglia derived from white matter from MS cases showed the same profile, but with significantly increased expression of CD14, suggesting a higher proportion having an M1 phenotype [30]. Further confirming the limited M1 phenotype of freshly isolated microglia, when these cells were put into culture they exhibited limited responses to LPS/IFN-γ [30]. By comparison, similarly prepared and cultured microglia could mount strong M2a and M2c responses when treated with IL-4 or glucocorticoid, respectively [6, 30]. In these studies, it was illustrated that human brain microglia cultured for up to 4 days showed progressive increased expression of CD14 [6, 31], while measurements of Toll-like receptor TLR-4 or IFN-γ receptor expression did not show this response. In a gene expression profiling study, we have shown that human microglia in culture demonstrated an acute M1-like response to 2 μM aggregated Aβ(1–42) after 24 hours of treatment [33]. Many of the genes associated with M2 responses or phagocytosis were downregulated. An alternative approach to profiling microglia that is being explored involves laser capture microdissection (LCM) of microglia from brain tissue sections followed by gene expression profiling. LCM has been widely used to dissect out discrete populations of neurons, astrocytes and endothelial cells from intact tissue sections; these techniques are also applicable for microglia [5]. One advantage of expression profiling methods for characterizing microglia is that they permit studies of genes not readily amenable to antibody staining (e.g., soluble chemokines/cytokines).

The morphology of HLA-DR immunoreactive microglia, with their close interaction with disease-associated cellular structures in AD brains, suggests a classical activation phenotype. It is noticeable that only a subset of these microglia in AD brains appears to be phagocytic. Contrary to what is observed in PD, where phagocytosis by microglia of neuromelanin can be seen, or in MS, with phagocytosis of myelin, observing microglia phagocytosis of Aβ in AD tissue sections is not as common even though there is a microglial “response” to plaques. Differences in microglia properties have been defined in neuropathology studies of subjects that had been immunized with amyloid peptides as part of experimental treatments [20, 29]. It has been suggested that classically activated microglia actually show reduced phagocytosis. The Aβ immunization strategy highlights this with the need for Aβ to be complexed with antibodies to promote polarization to a phagocytic phenotype. The interaction of immune complexes with primed microglia is a feature of M2b activated microglia.

An unanswered question is whether there are M2a or other alternatively activated microglia in AD or PD brains. There have been no reports demonstrating localization of markers of M2a alternative activation markers on microglia in AD or PD brains, even though increased expression of M2a markers CD206 and arginase-1 could detected by mRNA expression in AD tissue samples [26, 34]. There is a need to be able to distinguish by location between macrophages, which can be abundant in blood vessels of the brain, and microglia in the neuropil. CD200R, a new marker for M2a activation for microglia or macrophages, could also not be immunolocalized to microglia in AD tissue samples, even though mRNA expression was detectable [35]. CD200R immunoreactivity could only be detected in vascular macrophages in normal or MS tissue [21, 36]. Similarly, expression of CD200R in ex vivo isolated microglia was virtually undetectable even though expression was readily detectable in similarly isolated macrophages [36, 37]. These findings suggest that microglia with M2a phenotype are rare in AD brains. The identification of the most suitable marker for M2a in human tissues is still undecided. In MS tissues, immunoreactivity on lesion-associated microglia was observed for CCL22 and CD209, but not for CD206 [21].

## Possible immune phenotype markers besides MHC-II and IBA-1

Table 1 presents some of the markers that have been reported in (primarily) human AD tissues to identify different features of microglia. Specific features of several of these markers are discussed below.

### CD14

CD14 (LPS receptor) has been used in flow cytometric analyses to discriminate degrees of activation in macrophages and microglia. To some, CD14 has been considered a constitutive macrophage marker that all microglia will be expressing; however, in populations of monocytes/microglia, high or low expression of CD14 has been useful to define levels of activation [6]. Surprisingly, there have been only two studies that characterize cellular localization of CD14 expression in microglia in human AD-affected brains [15, 16]. CD14 antibody stains blood monocytes in brain vessels abundantly, and a very small percentage of plaque-associated microglia.

### Immunoglobulin Fc receptors (CD16, CD32 and CD64)

There are multiple types of the FcγR family expressed by macrophages and microglia. These include CD64 (FcγRI), CD16a (FcγRIIIA) and CD16b (FcγRIIIB) that activate proinflammatory signaling, and CD32a (FcγRIIA) and CD32b (FcγRIIB) that activate inhibitory signaling. All of these receptors can be considered phagocytic. Increased expression of these receptors has been associated with acquisition of the M2b phenotype, which has overlap with, but distinct differences from, gene profiles of M1 and M2a [26]. Microglia expressing CD16, CD32 and CD64 have been described in AD brains with increased levels of expression in pathology-associated microglia [13, 38].

### CD45

The usefulness of phenotyping human brain microglia for CD45 (leucocyte common antigen) is unclear because this marker appears to identify all microglia in human brains, but there are increased levels in AD pathology-associated microglia [39]. Phenotyping of microglia for CD45 immediately after their isolation from human brain tissues showed lower levels than in macrophages, while white matter microglia had higher CD45 expression than gray matter cells, with microglia from MS brains having increased CD45 expression compared with control brains [39, 40]. Treatment of ex vivo isolated microglia with LPS, IFN-γ, IL-4 or dexamethasone—the stimuli for inducing M1, M2a or M2c polarity, respectively—did not induce CD45 expression.

### CD68

One of the most useful and descriptive markers for microglial function has been CD68 (macrosialin in mice). This protein is localized to the lysosomal membrane in microglia and monocytes, and is upregulated in actively phagocytic cells [20]. Both M1 polarized and M2 polarized microglia/macrophages can express CD68 [11].

### Ferritin

Antibodies to l-ferritin selectively identify classes of microglia in human brain tissue sections [41]. Ferritin is the most abundant carrier protein for iron in the brain. Increased expression of ferritin associated with increased iron uptake seems associated with microglia undergoing proinflammatory responses and reactive oxygen species production, which is dependent on iron. Ferritin has been used to describe a dystrophic and degenerating phenotype of microglia, but their immune phenotype is unclear [42].

### Cyclooxygenase-1 and cyclooxygenase-2

Cyclooxygenase (Cox)-1 and Cox-2 have been favored targets for anti-inflammatory therapy for AD, but with limited beneficial results. Although associated with inflammation, Cox-2 has not been observed in microglia in AD brains, while Cox-1 has been localized to microglia associated with plaques [43]. Increased expression of Cox-1 in human macrophages has been characterized as an M2a response [11].

### CD33

CD33 (Siglec-3) is a sialic acid activated receptor whose expression is generally restricted to myeloid cells. CD33 is expressed on microglia in human brains [44, 45]. Intense interest in this marker has come from the identification of a single nucleotide polymorphism (SNP rs3865444) adjacent to the CD33 coding region being associated with altered risk of developing AD (odds ratio −0.89). Possession of the protective SNP results in reduced levels of CD33 in human brains [44, 45]. Our findings suggest that CD33 is constitutively expressed on microglia with increased levels on hypertrophic microglia. The regulation of CD33 expression by microglia has not been defined so it is not clear whether this marker reflects the M1 or M2 phenotype. Owing to anti-inflammatory signaling properties, CD33 and related sialic acid binding receptors have been suggested to polarize microglia towards an M2 phenotype; however, our in vitro data with human microglia showed that CD33 mRNA expression was not induced by LPS or IFN-γ (M1 stimuli) or by IL-4 (M2a stimulus) [45].

### Triggering receptor expressed by myeloid cells-2

Another microglial protein that has been associated with altered risk of AD is the triggering receptor expressed by myeloid cells-2 (TREM-2), where the mutation R47H in the coding sequence (SNP rs75932628) is associated with increased risk of AD. We have observed that there is increased expression of nonmutated TREM-2 on plaque-associated microglia in AD brains, with increased levels in AD tissues compared with control tissue [46]. There are insufficient data to indicate whether increased TREM-2 can be considered as a M1 or M2a activation marker in human microglia. Functionally, the marker appears similar to M2a anti-inflammatory receptors.

### CCL22 (monocyte-derived chemokine)

One recent study which defined CCL22 as an M2a marker by showing increased secretion by cultured human microglia in response to IL-4 also demonstrated microglia associated with MS lesions as having CCL22 immunoreactivity. These microglia were also immunoreactive for HLA-DR [21].

## Should we be defining an “M3” phenotype?

A potential limitation to the M1 or M2 immune phenotyping scheme is that it seems to omit microglia undergoing cell division as a response to macrophage colony-stimulating factor CSF-1 or the recently identified IL-34. Cell division by microglia can be considered an ongoing feature of microglia in pathology-rich areas, and is required to replace these short-lived cells. Both cytokines signal through the same macrophage/microglia receptor (CSF-1R) and not only induce cell division of microglia and critically affect their development, maturation and survival [47]. Examination of CSF-1R immunoreactivity of microglia in humans is limited to a single study that demonstrated a certain level of expression in control brains with increased expression in AD brains, especially in microglia accumulated around plaques [23]. Recent findings suggest that macrophage colony-stimulating factor-treated microglia have neither an M1 or M2a polarization state [48], although other works have classified CSF-1R signaling as similar to M2a activation [10].

## Conclusions

Until we have the reagents to detect microglial activities in human brain tissues associated with immune phenotypes and function, for example the production of reactive oxygen species or secretion of cytokines, a panel of antigenic markers that can be used to assign phenotype and function to identified microglia would be useful. Many of the initial papers describing microglial markers are now dated, but certain of these need to be reexamined in the context of understanding current concepts of phenotype. In addition, more recent papers describing profiling of human macrophages and microglia have identified new markers that can be applied to immunohistochemistry of diseased brain tissue if suitable antibodies are available.

**Fig. 2:**
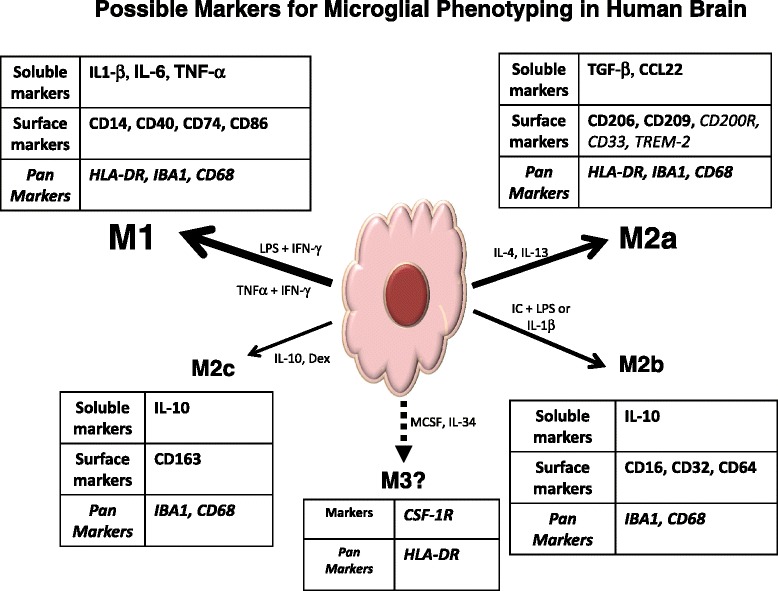
Possible markers for microglial phenotyping in human brains. Scheme to illustrate different markers that could be used for identifying different microglial phenotypes in human brains. Data show some markers that have been applied from more than one study. We include markers whose function is suggestive of polarity (e.g., CD200R, CD33 and TREM-2) but not proven with published data for human microglia. *CCL* C–C chemokine ligand, *CD200R* CD200 receptor, *CSF-1R* colony-stimulating factor-1 receptor, *IBA-1* ionized calcium binding adaptor molecule-1, *IFN-γ* interferon gamma, *IL* interleukin, *LPS* lipopolysaccharide, *TGF* transforming growth factor, *TNF* tumor necrosis factor, *TREM-2* triggering receptor expressed by myeloid cells-2

Figure 2 illustrates some of the markers that have been used to describe microglia and others that can be used to define their different phenotypes. The literature does have some conflicting results for certain of these markers; in some cases, there is discrepancy between mRNA and protein data.

Some of the publications on microglia markers have come about as a result of the availability of antibodies that react well with fixed human brain tissues. Many antibodies available to these discussed markers do not react with antigens fixed according to routine pathology procedures.

Although there are other candidates that can be reexamined, we propose that CD14, the LPS receptor, has been underappreciated as a functional activation marker of microglia. CD14 seems to be a sensitive marker for primed microglia, because levels of expression in microglia newly isolated from the human brain [6, 30–32] are very low but are higher in diseased MS brains [30]. High levels of CD14 in macrophages correspond to immune activation, but expression is suppressed in the brain; however, with the appropriate antibody we are reexamining the identification of CD14-positive microglia (in preparation, Walker and Lue, 2015), where positive reactivity must have some specific activation state. It was already known that only a small subset of microglia around plaques was immunoreactive in AD brains [16]. Freshly isolated microglia from nondiseased brain show very weak response to LPS, probably due to low levels of CD14 and TLR4 expression. Increased pattern expression of CD14 in microglia in diseases could be informative to describing M1 activated microglia.

In conclusion, it may be a hard task to bring together the fundamental findings of immunologists and cell biologists, on one hand, with the approaches of practicing neuropathologists, on the other, but the potential could be to make discoveries on how and where inflammation is actually causing neurodegeneration (or neuroregeneration). From such observations, new disease-modifying molecular targets can evolve.

## Note

This article is part of a series on *Innate Immunity*, edited by Donna Wilcock. Other articles in this series can be found at http://alres.com/series/innateimmunity

## Abbreviations

AD: Alzheimer’s disease
AIF-1: Allograft inflammatory factor-1
Aβ: Amyloid beta peptide
CCL: C–C chemokine ligand
CCR: C–C chemokine receptor
CD: Cluster of differentiation
CD200R: CD200 receptor
Cox: Cyclooxygenase
CSF-1R: colony-stimulating factor-1 receptor
CXCL: C–X–C chemokine ligand
DLB: Dementia with Lewy bodies
FcγR: Immunoglobulin Fc gamma receptor
FTD: Frontal temporal dementia
IBA-1: Ionized calcium binding adaptor molecule-1
IFN-γ: Interferon gamma
IL: Interleukin
ILBD: Incidental Lewy body disease
LCM: Laser capture microdissection
LPS: Lipopolysaccharide
MHC-II: Major histocompatibility class II
MS: Multiple sclerosis
MSR: Macrophage scavenger receptor
NADPH: Nicotinamide adenine dinucleotide phosphate
PD: Parkinson’s disease
SN: Substantia nigra
SNP: Single nucleotide polymorphism
TGF: Transforming growth factor
TLR: Toll-like receptor
TNF: Tumor necrosis factor
TREM-2: Triggering receptor expressed by myeloid cells-2
